# Post-bariatric body contouring surgery: analysis of complications in 180 consecutive patients

**DOI:** 10.1590/0100-6991e-20202638

**Published:** 2021-07-10

**Authors:** WILSON CINTRA, MIGUEL LUIZ ANTONIO MODOLIN, DIEGO RICARDO COLFERAI, RODRIGO ITOCAZO ROCHA, ROLF GEMPERLI

**Affiliations:** 1 - Hospital das Clínicas da Faculdade de Medicina da Universidade de São Paulo, Divisão de Cirurgia Plástica - São Paulo - SP - Brasil

**Keywords:** Obesity, Morbid, Bariatric Surgery, Body Contouring/adverse effects, Postoperative Complications/surgery, Obesidade Mórbida, Cirurgia Bariátrica, Contorno Corporal/efeitos adversos, Complicações Pós-Operatórias/cirurgia

## Abstract

**Introduction:**

bariatric surgery is the main treatment for cases of severe obesity and body contour surgery to correct body dysmorphia resulting from weight loss. However, these procedures are associated with a significant number of postoperative complications.

**Objective:**

this study aims to analyze complications in post-bariatric patients undergoing body contour surgeries and correlating them with the age and BMI of these patients.

**Methods:**

the current study is a retrospective study evaluating 180 consecutive patients undergoing body contour surgery after bariatric surgery within a period of three years (2014-2016). Data such as age, gender, Body Mass Index before bariatric and plastic surgeries, type of surgery performed and complications were collected, and correlated the age as well as the BMI of the patients in the pre-bariatric (PB) and pre-plastic (PP) periods with the complications presented.

**Results:**

of the 180 patients evaluated, 91.7% were females (n = 165), and the mean age was 46.3 ± 1.7 years. The most performed surgery was abdominoplasty (48.9%), followed by mammaplasty (21.1%). Some complications occurred in 26.1% of the patients with partial dehiscence (40.4%) and seroma (14.9%) being the most frequent. Patients who presented complications had a higher mean age (50.8 years) than those who presented with no complications, and major complications accounted for 2.7% of the sample.

**Conclusions:**

a statistically significant number of surgeries progressed without complications and, when they occurred, there were minor complications in most of the sample. Complications were more frequent in older patients with some of them having a BMI over 30 Kg/m^2^.

## RESUMO

**Introdução:**

a cirurgia bariátrica é o principal tratamento para os casos de obesidade grave e a cirurgia de contorno corporal trata a dismorfia corporal resultante desta perda de peso. No entanto, estes procedimentos estão associados a um número significativo de complicações pós-operatórias.

**Objetivo:**

analisar as complicações maiores e menores que ocorreram nos pacientes pós-bariátricos submetidos a cirurgias reparadoras do contorno corporal e correlacioná-las com idade e IMC destes pacientes.

**Métodos:**

estudo retrospectivo que avaliou 180 pacientes consecutivos submetidos a cirurgia de contorno corporal, após cirurgia bariátrica, no período de 3 anos (2014-2016). Foram coletados dados como idade, gênero, IMC pré-bariátrica e pré-plástica, tipo de cirurgia realizada e complicações, correlacionando a idade e o IMC dos pacientes nos períodos pré-bariátrica (PB) e pré-plástica (PP) com as complicações apresentadas.

**Resultados:**

dos 180 pacientes avaliados, 91,7% eram do gênero feminino (n=165) e a idade média foi de 46,3 ± 1,7 anos. A cirurgia mais realizada foi abdominoplastia (48,9%), seguida da mamoplastia (21,1%). Complicações ocorreram em 26,1% dos pacientes, sendo deiscência parcial a principal (40,4%) seguida de seroma (14,9%). Pacientes com complicações apresentaram média etária maior (50,8 anos) que os sem complicações, sendo que as complicações maiores ocorreram em 2,7% da amostra.

**Conclusões:**

a maioria estatisticamente significante das cirurgias cursou sem complicações e, quando ocorreram, foram complicações menores na maior parte da amostra. As complicações foram mais frequentes nos pacientes de idade mais avançada, dos quais alguns com IMC maior que 30kg/m^2^.

## INTRODUCTION

Obesity is currently a global epidemic, accounting for reduction in life expectancy, increased mortality, worsening in quality of life, and high costs to public health^1^. Worldwide, 1.9 billion people over the age of 18 are overweight; of these, 650 million are obese. The prevalence of overweight and obesity in children and adolescents has increased dramatically in the last 30 years, rising from 4% in 1975 to 18% in 2016^2^. In Brazil, the prevalence of obesity rose from 11.8% in 2006 to 19.8% in 2018. The greatest increase occurred in adults aged between 24 and 44 years, the female population surpassing the male one^3^.

Clinical and surgical methods have been used for obesity treatment. Clinical treatment is effective for most obese patients, but in case of the severely obese, bariatric surgery is the most effective method. Thus, an increasing number of patients are submitted to this surgical modality, which provides rapid and intense weight loss, but results in functional and aesthetic body sequelae^4^. Such changes correspond to what is conventionally called dysmorphia, characterized by fatty accumulations predominantly in the arms, breasts, abdomen, and thighs^5^. 

To facilitate mobility and personal hygiene, improve the overall appearance, and the psychological profile, these emaciated patients undergo operations of the body contour^5^ ^,^ ^6^. However, these procedures may display higher complication rates due to the association with obesity^7^ ^-^ ^12^, as well as being riskier among elderly patients^9^.

Surgical complication is defined as any deviation that interferes with the expected recovery. In general, complications are associated with patient’s general condition, the magnitude of the procedure, or inadequate surgical technique^6^ ^,^ ^8^ ^,^ ^10^. They are classified as major and minor. Major complications are those that require surgical reintervention or increase the length of hospital stay; minor ones are amenable to outpatient treatment, through small procedures such as punctures, drainages, or dressings^7^ ^-^ ^11^.

## OBJECTIVE

The aim of this study is to analyze the major and minor complications that occurred in patients after bariatric surgery who underwent body contouring repair operations and to associate them with age and Body Mass Index (BMI).

## METHODS

This is a retrospective study carried out by analyzing the medical records of patients seen at the Plastic Surgery and Burns Division.

In the three-year period, between January 2014 and December 2016, 180 patients were operated on. Seven of these patients underwent associated operations, totaling 187 procedures.

We included patients of both sexes, aged between 18 and 69 years, who had undergone bariatric surgery, lost weight, and had a stable weight for a minimum period of 12 months. Exclusion criteria were smoking, alcohol consumption, and use of illicit drugs. All patients underwent clinical and laboratory evaluations that confirmed their aptitude for the surgical procedure. Female patients were not in use of hormone replacement therapy or contraception. Psychological evaluation was performed in all patients, with the aim of assessing the emotional profile, aligning expectations, and informing about possible complications. All individuals signed an informed consent form and a photographic documentation authorization form.

The research project was presented to CAPPesq - Ethics Committee for Analysis of Research Projects at the Hospital das Clínicas, Faculty of Medicine, University of São Paulo, under registration number 11,819, and approved without restrictions. The project was also registered and approved by the Brazil CONEP Platform - National Research Ethics Commission, Ministry of Health, CAAE number 48112015.8.0000.0068.

We administered prophylactic antibiotic therapy with a first generation cephalosporin - cefazolin or cephalothin - to all patients. Every individual used compression stockings, intermittent massager for the lower limbs, and a thermal blanket during the operation. As an additional preventive measure against thromboembolic events, we administered low molecular weight heparin to all patients.

We collected demographic and sample characterization data, such as age, sex, body mass index before bariatric surgery - pre-bariatric (PB) and before plastic surgery - pre-plastic (PP), type of operations performed, and complications. Complications were classified as major and minor and were compared with BMI and patient age.

The computed sample size, adopting a 0.10 null hypothesis, was of 108 participants. The results were subjected to statistical analysis. We used the Two Proportions Equality test to characterize the distribution of the relative frequencies (percentages) of qualitative variables, such as sex, operations, complications, as well as types and degree of complications. We used the ANOVA test to compare patients’ complications and age. To compare the mean BMI between the PB and PP times, we used the paired Student’s T test, since the patient was his/her own control. Finally, we used the Chi Square test to measure the degree of relationship between complications and BMI, both PB and PP.

## RESULTS

The sample consisted of 180 patients, with a mean age of 46.3 ± 1.7 years, ranging from 18 to 69. The predominant sex was female (91.7%).

There was a reduction in BMI from the PB to the PP periods, with an average evolving from 48.7 kg/m^2^ to 29.5 kg/m^2^ (p <0.001). There was a difference between sexes for BMI in both the PB and in PP periods, and the average for men was always higher than for women, as in PP, when we observed 34.3 kg/m^2^ and 29.1 kg/m^2^, respectively (p <0.001).

The patients (n = 180) underwent 187 surgical procedures, seven of them undergoing associated operations. The most performed procedures were abdominoplasty (52.2%) and mastoplasty (22.8%), as shown in Table 1.

**Table 1:** Distribution of procedures.

Surgery	N	%	p-value
Abdominoplasty	94	52.2%	Ref.
Mamoplasty	41	22.8%	<0.001
Cruroplasty	21	11.7%	<0.001
Brachioplasty	19	10.6%	<0.001
Liposuction	5	2.8%	<0.001
Circumferential abdominoplasty	4	2.2%	<0.001
Ritidoplasty	2	1.2%	<0.001
Gynecomasty	1	0.6%	<0.001
Total	187		

Complications were present in 47 patients, corresponding to 26.1% (p <0.001), the main ones being partial dehiscence (40.4%) and seroma (14.9%), as recorded in Table 2.

**Table 2:** Types of complications.

Type complications	N	%	p-value
Dehiscence	19	40.4%	Ref.
Seroma	7	14.9%	0.006
Hypertrophic scar	5	10.6%	<0.001
Epitheliolysis	3	6.4%	<0.001
Hematoma	3	6.4%	<0.001
Surgical wound (SW) infection	4	8.4%	<0.001
Abscess	1	2.1%	<0.001
Asymmetry	1	2.1%	<0.001
Asymmetry, hematoma	1	2.1%	<0.001
SW Infection, dehiscence, lymphorrhea	1	2.1%	<0.001
Necrosis	1	2.1%	<0.001
Neuralgia	1	2.1%	<0.001

Major complications occurred in five patients, corresponding to 10.6%, or 2.7% of the sample (Table 3).

**Table 3:** Degree of complications.

Degree	N	%	p-value
Major	5	10.6%	<0.001
Minor	42	89.4%	

Most patients had no complications, and when they did occur, they were minor ones (p <0.001), as shown in Tables 4 and 5.

**Table 4:** Distribution of complications by type of surgery.

	Without complication		With complication		p-value
	N	%	N	%	
Abdominoplasty	69	73.4%	25	26.6%	<0.001
Mamoplasty	32	78.0%	9	22.0%	<0.001
Brachioplasty	14	73.7%	5	26.3%	0.004
Cruroplasty	15	71.4%	6	28.6%	0.005
Circumferential abdominoplasty	1	25.0%	3	75.0%	0.157
Liposuction	5	100.0%	0	0.0%	0.002
Ritidoplasty	2	100.0%	0	0.0%	0.157
Gynecomasty	0	0.0%	1	100.0%	0.157

**Table 5:** Distribution by complication degree and type of surgery.

	Minor		Major		p-value
	N	%	N	%	
Abdominoplasty	22	88.0%	3	12.0%	<0.001
Mamoplasty	8	88.9%	1	11.1%	<0.001
Brachioplasty	5	100.0%	0	0.0%	0.002
Cruroplasty	6	100.0%	0	0.0%	<0.001
Circumferential abdominoplasty	2	66.7%	1	33.3%	0.414
Gynecomasty	1	100.0%	0	0.0%	0.157

 There was a statistically significant difference in complications regarding age, since patients with complication were, on average, 50.8 years old, as opposed to 44.7 years on average for those with no complications (p = 0.003), as described in Table 6. 

**Table 6:** Complication versus Age.

Complication		Mean	Median	Standard deviation	CV	Min	Max	N	CI	p-value
Age	With complication	50.8	52	10.8	21%	19	67	47	3.1	0.003
	Without complication	44.7	43	11.9	27%	18	69	133	2.0	

CV - coefficient of variation; CI - confidence interval.

Of the 180 patients, 27 were aged over 60. Of these, 12 (44%) had complications. Among 27 patients, 13 had a BMI > 30 kg/m^2^, and seven presented with complications.

 We observed no statistical relationship between complications and BMI (Table 7).

**Table 7:** Complications versus BMI.

		With complication		Without complication		Total		p-value
		N	%	N	%	N	%	
PB BMI	<50	24	51%	86	65%	110	61%	0.088
	>50	23	49%	46	35%	69	39%	
PP BMI	<30	25	53%	90	68%	115	64%	0.066
	>30	22	47%	42	32%	64	36%	

## DISCUSSION

The literature has no studies with significant samples mainly focusing on complications in patients undergoing operations for body contouring after major weight loss^7^ ^,^ ^11^ ^,^ ^12^.

The increase in bariatric procedures and the resulting deformities of the body contour, called dysmorphias, led to an increase in the number of patients after bariatric surgery seeking plastic surgery for physical and emotional rebalancing. With the popularization of plastic surgery and the improvement of operative techniques, the treatment of these patients has become more effective, achieving results that improve quality of life^12^ ^-^ ^16^.

Currently, the preferred option is for single procedures, since associated operations display higher complications rates^17^. Our sample consisted of 180 patients who underwent 187 procedures, that is, only seven patients underwent associated operations.

 In agreement with other publications, the female sex was predominant (91.7%). The average age was 46.3 ± 1.7 years, and the most performed operations were abdominoplasty (52.2%) and mastoplasty (22.8%).

 Complications are unfavorable and unexpected postoperative situations, which interfere with surgical recovery and modify the expected result. The study evaluated the occurrence of 47 complications, that is, in 26.1% of the cases. As mentioned in the introduction, complications can be major or minor; we observed a predominance of minor ones. Only five patients (2.7%) had major complications, requiring prolongation of the hospital stay, readmission, or surgical reintervention^18^. 

Although the literature stresses that patients with BMI greater than 30 kg/m^2^ have greater incidence of postoperative complications^7^, we did not observe this relationship (Table 7).

However, some data coincide, opposing an interpretation of mere coincidence. Of the 27 patients over the age of 60, 13 had a BMI greater than 30 kg/m^2^, and of these, seven had complications. Such a condition (age over 60 and BMI over 30 kg/m^2^) deserves to be taken into consideration, as older individuals display alterations in collagen synthesis, deposition and remodeling^19^ ^,^ ^20^. In addition, obese patients, therefore with a BMI greater than 30 kg/m^2^, have metabolic and inflammatory changes^21^ ^,^ ^22^, which, together with surgical trauma, can be associated with complications. In isolation, these alterations are compromising, and when combined, they can determine major complications. A most significant statistical power would perhaps be achieved with an adequate sample size, something we did not perform, which is the major limitation of our study.

## CONCLUSIONS

Most of the patients had no complications, and when occurred, these were minor, and more frequent among older patients (average age 50.8 years). There was no association between complications and BMI.
